# Supplementary figures and images for: Alpinetin protects against iron overload related osteoarthritis via NRF2/HO-1 pathway
Source: PLoS One. 2025 Jun 2;20(6):e0317930. doi: 10.1371/journal.pone.0317930 (PMC12129196; doi:10.1371/journal.pone.0317930)

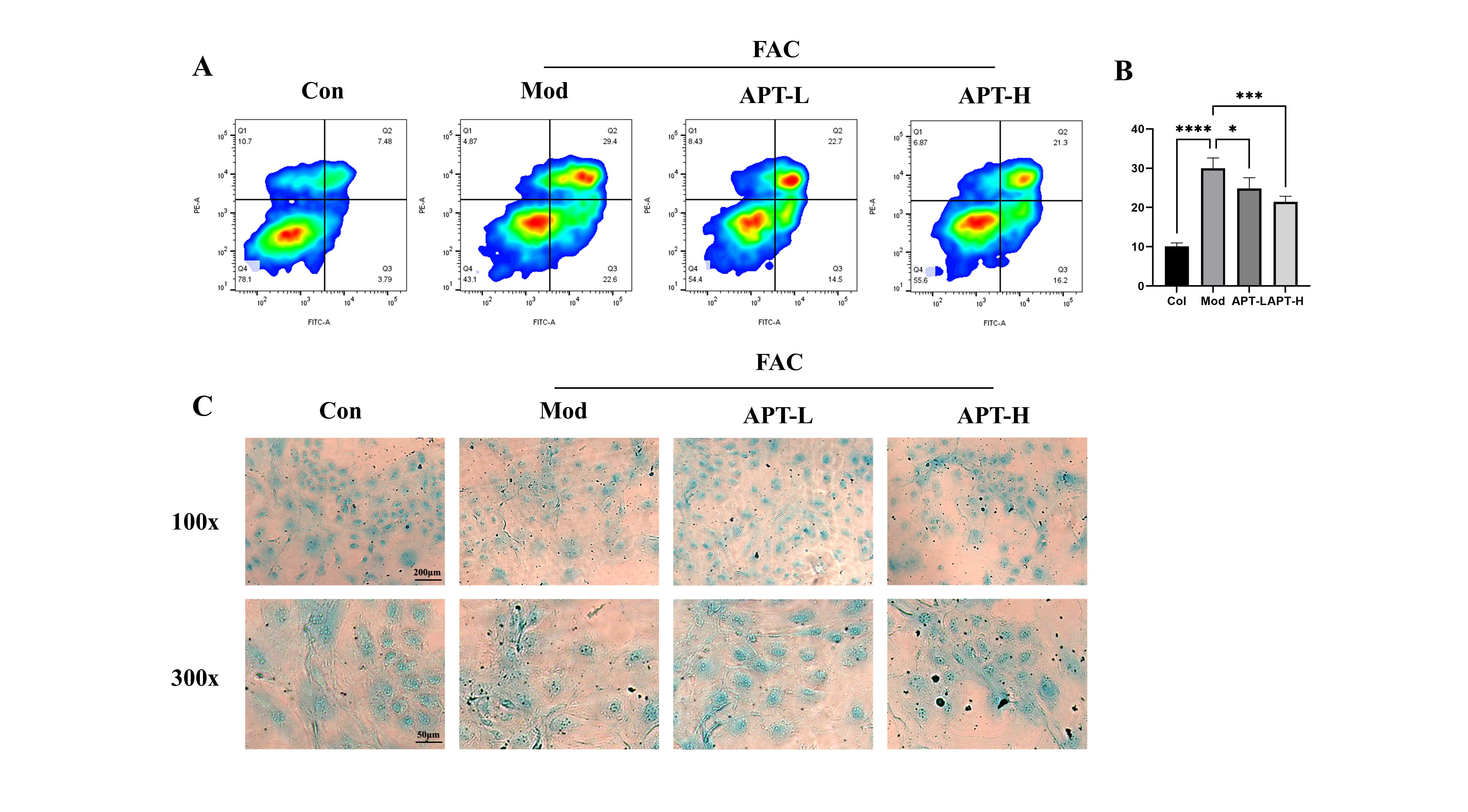

Supplement: S1 Fig — Chondrocyte apoptosis assay in adult mice (A,B); P2 Alisin blue staining of adult mouse chondrocytes (C). (TIF) [file pone.0317930.s001.tif]

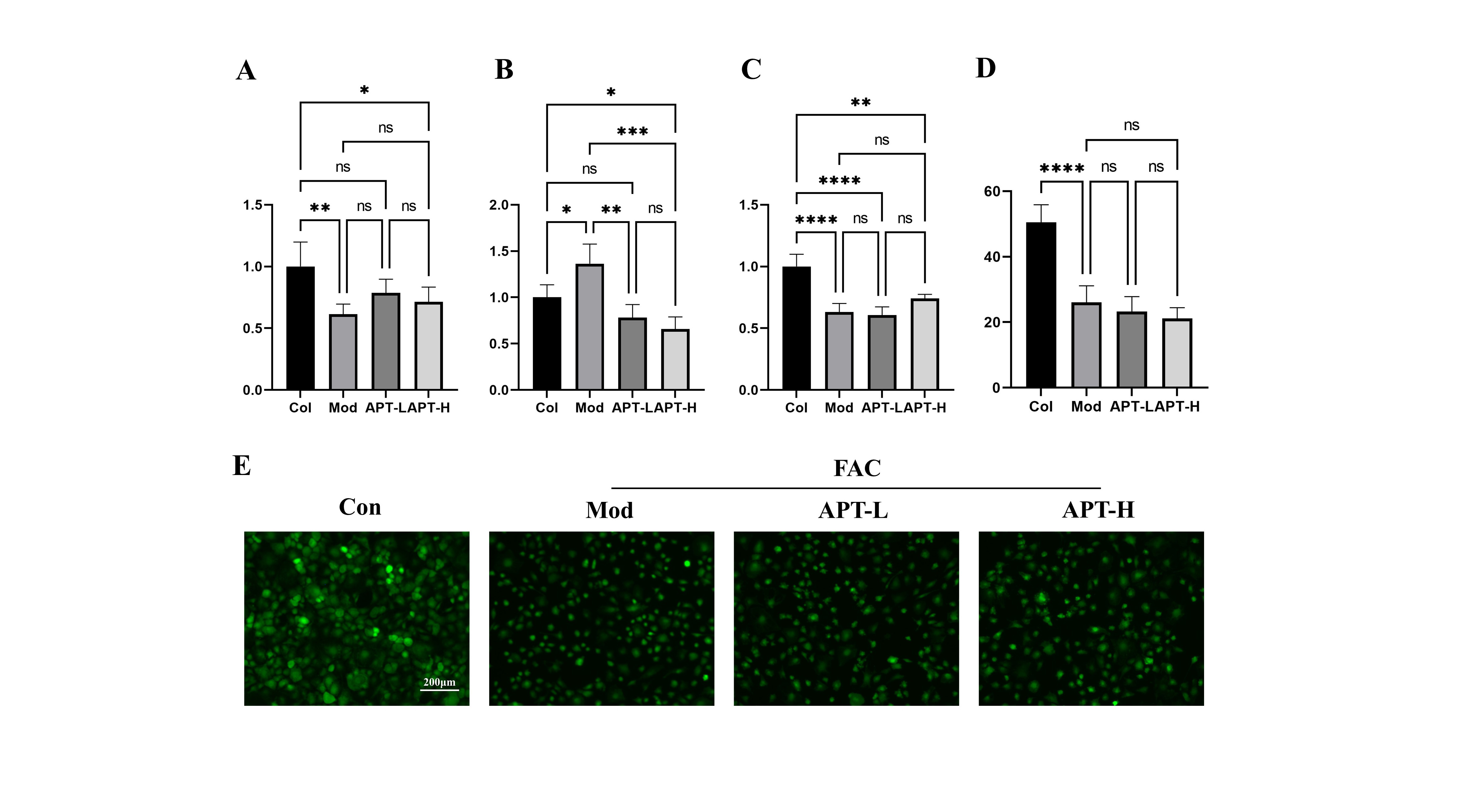

Supplement: S2 Fig — PCR results of DMT1 (A); PCR results of TFR1 (B); PCR results of FPN(C); Fluorogram and fluorescence quantification of calcineurin (D,E)。 (TIF) [file pone.0317930.s002.tif]
