# Supplementary material for: Alpinetin protects against iron overload related osteoarthritis via NRF2/HO-1 pathway
Source: PLoS One. 2025 Jun 2;20(6):e0317930. doi: 10.1371/journal.pone.0317930 (PMC12129196; doi:10.1371/journal.pone.0317930)
Supplement: Attachment — Submitted filename: Table 1.doc [file pone.0317930.s003.doc]

Table 1 RT-qPCR primer sequence

| PRIMERS NAME | FORWARD (5′-3′) | REVERSE (5′-3′) |
| --- | --- | --- |
| 18s | TGGTTGCAAAGCTGAAACTTAAAG | AGTCAAATTAAGCCGCAGGC |
| Col II | GCTGGTGAAGAAGGCAAACGAG | CCATCTTGACCTGGGAATCCAC |
| MMP3 | CTCTGGAACCTGAGACATCACC | AGGAGTCCTGAGAGATTTGCGC |
| Bax | AGGATGCGTCCACCAAGAAGCT | TCCGTGTCCACGTCAGCAATCA |
| NRF-2 | CAGCATAGAGCAGGACATGGAG | GAACAGCGGTAGTATCAGCCAG |
| HO-1 | CACTCTGGAGATGACACCTGAG | GTGTTCCTCTGTCAGCATCACC |
